# Validation of an artificial intelligence-based drawing platform against the Clock Drawing Test for cognitive screening: a comparative study between Alzheimer's disease and healthy controls

**DOI:** 10.1093/fampra/cmag066

**Published:** 2026-08-25

**Authors:** Ülkü Sur Ünal, Halil Alper Karabaş, Aybora Yadigar, Ahsen Simanur Turnalı, Elifnur Demir

**Affiliations:** Department of Family Medicine, Marmara University School of Medicine, Istanbul 34854, Türkiye; Marmara University School of Medicine, Istanbul 34854, Türkiye; Marmara University School of Medicine, Istanbul 34854, Türkiye; Marmara University School of Medicine, Istanbul 34854, Türkiye; Marmara University School of Medicine, Istanbul 34854, Türkiye

**Keywords:** Alzheimer’s disease, mental status and dementia tests, artificial intelligence, primary health care, diagnostic screening programmes, nursing homes

## Abstract

**Background:**

Traditional manual Clock Drawing Tests (CDTs) are widely used for cognitive screening but are constrained by human subjectivity and alphanumeric literacy or cultural barriers.

**Objective:**

This study validated an open-source artificial intelligence (AI)-based drawing platform utilizing universal, pre-linguistic Lea symbols against manual CDT metrics for cognitive screening and differentiating Alzheimer's disease (AD) from healthy controls (HCs).

**Methods:**

In this cross-sectional validation study, 111 participants aged ≥65 years, 55 clinically diagnosed AD patients and 56 HCs, were evaluated. Participants completed manual CDTs (6-point and 10-point scoring) and the AI-based drawing task on a tablet. Diagnostic accuracy was evaluated using receiver operating characteristic curve analysis, Cohen's kappa (*κ*), and multivariable logistic regression adjusted for age and gender.

**Results:**

The AI model demonstrated clear diagnostic accuracy (area under the curve = 0.868), with overall accuracy comparable to the 10-point manual scale. An optimal diagnostic cut-off of <27.00 points, identified in this study, achieved 83.6% sensitivity and 75.0% specificity, demonstrating substantial agreement with clinical diagnosis (*κ* = 0.586, *P* < 0.001). Crucially, the AI-based drawing platform flagged 14 HCs (25.0%) as at-risk who were classified as intact by conventional manual CDT scales. Multivariable regression confirmed the AI score as a powerful independent predictor of AD status after adjusting for demographics (odds ratio: 0.940, 95% confidence interval: 0.904–0.979, *P* < 0.001).

**Conclusion:**

The AI-based drawing platform provides an objective, automated alternative to traditional CDTs. Its sensitivity to subtle visuoconstructional variations positions it as an effective, low-cost triage tool to optimize early neurological referrals in primary care.

Key messages

- Artificial intelligence platform matches 10-point Clock Drawing Test accuracy (area under the curve = 0.868) in Alzheimer's screening.
- Automated digital scoring removes human observer bias in cognitive testing.
- Exploratory cut-off (<27.00) yields 83.6% sensitivity and 75.0% specificity.
- Flagged 25% of controls as at-risk, warranting closer follow-up and evaluation.
- Offers an objective, scalable tool for rapid cognitive triage in primary care.

## Introduction

Objective and accessible screening metrics are essential for effective cognitive screening and risk stratification of neurodegenerative disorders. In primary care and routine clinical settings, the Clock Drawing Test (CDT) remains a foundational tool for evaluating visuoconstructional and executive functions [1, 2]. However, traditional pen-and-paper CDT protocols depend heavily on alphanumeric literacy and abstract time concepts, making standard clock-face evaluations less reliable for populations with limited formal education or cultural and language barriers [3, 4]. Language-based tests lose severe sensitivity (dropping to 46%) in poorly educated older adults, whereas non-verbal drawing tasks overcome these literacy barriers [5]. Additionally, traditional manual CDT scoring may be less sensitive to mild cognitive impairment (MCI), as it may not fully capture subtle variations in visuoconstructional execution [6, 7]. Reiner *et al*. [8] showed standard CDTs have low specificity (45.6%) and high false-positives. Similarly, Bozikas *et al*. [9] demonstrated age-related drawing declines after 60 and 70 cause misclassifications.

Simplified tools like Lin *et al*.'s system compromise specificity, while complex rubrics yield high false-positive rates [10, 11]. Furthermore, protocol non-equivalence causes classification rates to fluctuate (9%–50%) based on the rater's scale, necessitating an automated framework [12, 13].

To address these limitations, a wide range of artificial intelligence (AI) driven tools, spanning from deep learning classifiers to digital drawing systems, have been developed to analyse drawing performance [14, 15]. Despite this technological diversity, digital adaptations of the CDT predominantly focus on the traditional alphanumeric clock face [16].

To bridge this methodological gap, this study evaluates an alternative application of an open-source AI-based drawing platform, adapting it to the universal, pre-linguistic symbols of the Lea Test paradigm (the circle, square, and apple). This task minimizes cultural and literacy biases by focusing strictly on visuoconstructional execution, making it highly practical for diverse and unselected primary care populations. Additionally, automated scoring provides an objective evaluation that addresses the constraints of traditional manual methods, such as inter-rater variability and consultation time limits.

Consequently, this methodological validation study aims to evaluate the diagnostic accuracy of this AI-based drawing platform against established 6-point and 10-point CDT scoring systems [17, 18]. By comparing clinically diagnosed Alzheimer's disease (AD) patients with cognitively healthy older adults, we examine the utility of this objective and accessible framework as a practical triage tool for rapid cognitive screening and clinical differentiation within primary care environments.

## Methods

### Study design and setting

This cross-sectional, methodological validation study evaluated the diagnostic accuracy of an AI-based drawing platform against the traditional CDT for AD. Approved and overseen by the Marmara University School of Medicine, Department of Family Medicine, data collection occurred between 1 November 2023, and 1 February 2024, with data verification and quality control processing extending to 1 May 2024.

### Participants and selection criteria

#### Sampling frame and target population

The baseline population comprised individuals aged 65 years and older registered in the Tuzla Education-Family Health Centre database (*N* = 574). Within this primary care pool, 73 patients with a clinical diagnosis of AD were identified using ICD-10 codes G30 and F00. Of these 73 individuals, 32 were institutionalized residents at the Tuzla Nursing Home. To expand clinical recruitment and reach the necessary sample size, eligible AD patients from a second long-term care facility in the Maltepe district were integrated into the clinical sampling frame.

#### Group allocation

The final study sample consisted of 111 participants divided into two size-matched cohorts:

- AD group (*n* = 55): Utilizing a total population sampling approach, all available and eligible AD patients within the two designated nursing homes were invited to participate. Ultimately, 55 patients (or their legally authorized representatives) provided informed consent and successfully completed the evaluation protocol.
- Healthy control (HC) group (*n* = 56): To establish the HC, the 73 diagnosed AD patients were excluded from the original database, leaving a subpopulation of 501 non-demented individuals. From this remaining pool, 56 cognitively healthy participants were enrolled via convenience sampling to serve as a baseline comparison group.

#### Sample size and power justification

An *a priori* sample size calculation was not performed due to the finite size of the accessible clinical population within the partner institutions; recruitment focused on maximum feasible enrolment during the study window. However, a *post-hoc* power analysis indicated that the final sample size of *N* = 111 (*n* = 56 HC and *n* = 55 patients with AD) provides a statistical power exceeding 80% (power = 0.80) to detect a large effect size (Cohen's *d* ≥ 0.55) in a two-tailed independent samples *t*-test, and moderate-to-strong correlations (*r* ≥ 0.35) using Spearman's rank correlation (*α* = 0.05).

### Data collection and assessment tools

All participants completed two parallel, single-session assessments:

- Traditional CDT: The manual CDT was administered to evaluate visuoconstructional and executive functions. To minimize scoring bias, drawings were independently evaluated using the Shulman 6-point and Sunderland 10-point scoring systems [17, 18].
- AI-based drawing assessment: In parallel, participants completed a drawing task on a tablet using a digital pen, where they were asked to reproduce three standardized Lea symbols (a circle, square, and apple). The drawing data were evaluated using an open-source, pre-trained Convolutional Neural Network architecture optimized for sketch recognition. The MobileNet-based pipeline converts stroke paths from vector to raster, scaling and centring them within a 256 × 256 pixel bounding box to standardize size and thickness. This deep learning model, trained on the comprehensive *Google* ‘Quick, Draw!’ dataset, processes the final drawing output by computing a structural confidence score based on a soft-max probability distribution. This score measures the geometric similarity and structural integrity of the participant's drawing relative to the high-dimensional validation weights of the target templates.

### Statistical analysis

Statistical analyses were performed using Jamovi (version 2.4). Data normality was verified via the Shapiro–Wilk test. Since AI drawing scores and manual CDT metrics exhibited non-normal distributions (*P* < 0.05), non-parametric methods were applied. Group differences between AD and HC cohorts were evaluated using the independent samples *t*-test for age, Mann–Whitney *U* test for digital and manual scores, and Pearson chi-square (*χ*^2^) test for gender distribution. Within-group associations were analysed using Spearman rank correlation (*ρ*).

The diagnostic accuracy of the metrics was quantified via receiver operating characteristic (ROC) curve analysis, with 95% confidence intervals (CIs) calculated using bootstrapping (1000 resamples) and cut-off thresholds determined by Youden's Index. Classification agreement with clinical diagnosis was evaluated using Cohen's kappa (*κ*). Finally, a multivariable binary logistic regression model [reporting McFadden's pseudo-*R*^2^, odds ratios (ORs), and 95% CIs] was constructed to predict AD status while adjusting for age and gender. Statistical significance was set at *P* <0.05.

### Ethical considerations

The study complied with the Declaration of Helsinki and was approved by the Marmara University Clinical Research Ethics Committee (Protocol: 09.2023.1420). Written or witnessed verbal informed consent was obtained from all participants or their legally authorized representatives prior to enrolment.

We used the STARD reporting guideline to draft this manuscript, and the STARD reporting checklist when editing, included in Supplement A [19].

## Results

### Baseline demographics and clinical characteristics

The study population comprised 111 participants, divided into the AD group (*n* = 55) and the HC group (*n* = 56). The AD group was significantly older than the HC group (*P* < 0.001), while gender distribution did not differ significantly between the cohorts (*P* = 0.064).

Cognitive performance across all evaluated metrics was significantly lower in the AD cohort compared to the HC cohort. The median AI drawing score was 3.67 for the AD group and 49.33 for the HC group (*P* < 0.001). Lower scores were also observed in the AD population for both traditional manual metrics: the 10-point Sunderland scale and the 6-point Shulman scale (all *P* < 0.001; Table 1).

**Table 1:** Baseline demographic and clinical characteristics of the study cohort .

Characteristics/variables	Healthy controls (*n* = 56)	Alzheimer's disease (*n* = 55)	Test statistic	*P*-value
Age (years) [mean ± SD]	69.98 ± 5.40	84.91 ± 6.53	*t* = −13.11	**<0.001**
Gender (female/male) [*n* (%)]	28 (50.0%)/28 (50.0%)	38 (69.1%)/17 (30.9%)	*χ^2^* = 3.44	0.064
AI drawing score [median (IQR)]	49.33 (26.58–66.67)	3.67 (0.00–16.00)	*U* = 2674.5	**<0.001**
CDT (10-point scale) [median (IQR)]	8.00 (5.75–9.00)	0.00 (0.00–2.00)	*U* = 2673.5	**<0.001**
CDT (6-point scale) [median (IQR)]	5.00 (4.00–6.00)	2.00 (1.00–3.00)	*U* = 2802.0	**<0.001**

Statistically significant *P*-values are highlighted in bold.

AI, artificial intelligence; CDT, Clock Drawing Test; IQR, interquartile range (25th–75th percentiles).

### Diagnostic accuracy and ROC curve analysis

**Figure 1:**
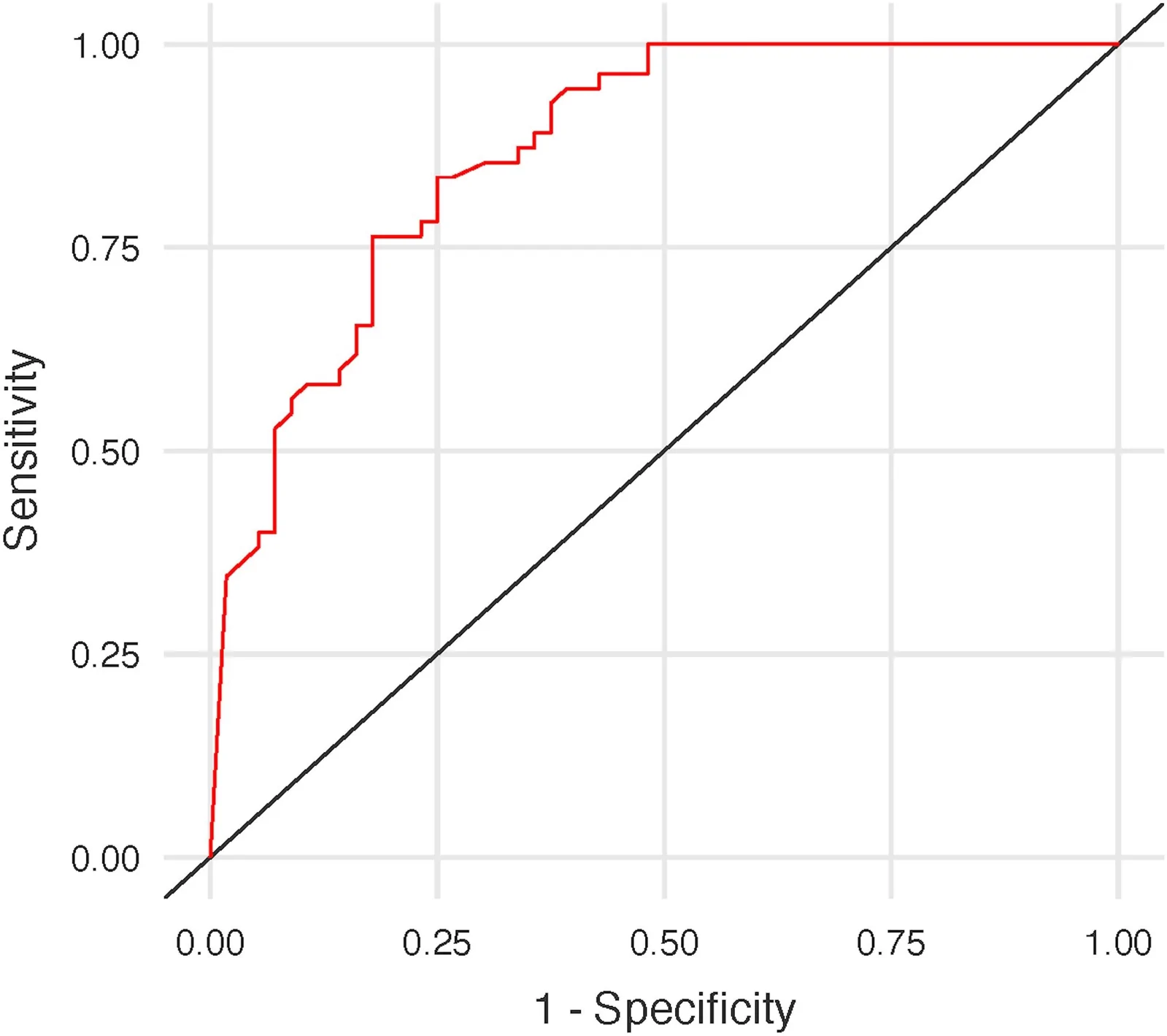
Receiver operating characteristic (ROC) curve evaluating the diagnostic capacity of the AI-based drawing platform for differentiating patients with Alzheimer's disease from healthy controls.

ROC analysis was used to assess the performance of each tool in differentiating AD patients from HCs (Fig. 1, Table 2). The AI-based drawing platform demonstrated a robust area under the curve (AUC = 0.868, 95% CI: 0.800–0.928), matching the classification performance of the traditional 10-point manual scale (AUC = 0.868, 95% CI: 0.792–0.934). The manual 6-point scale achieved the highest overall discriminative power (AUC = 0.910, 95% CI: 0.846–0.960).

**Table 2:** Receiver operating characteristic curve analysis and diagnostic cut-off parameters.

Predictive model	AUC	95% CI	Optimal cut-off	Sensitivity (%)	Specificity (%)
AI drawing score	0.868	[0.800–0.928]	≤27.00	83.6	75.0
CDT (10-point scale)	0.868	[0.792–0.934]	≤2.00	81.8	87.5
CDT (6-point scale)	0.910	[0.846–0.960]	≤3.00	83.6	89.3

95% CIs were computed via bootstrapping with 1000 resamples. Optimal cut-off thresholds were determined by maximizing Youden's Index.

AI, artificial intelligence; AUC, area under the curve; CDT, Clock Drawing Test, CI, confidence interval.

Based on Youden's Index, the optimal diagnostic cut-off for the AI drawing score was established at ≤27.00 points, yielding a sensitivity of 83.6% and a specificity of 75.0%. This automated classification also demonstrated strong mathematical alignment with the established clinical diagnosis (*κ* = 0.586, *P* < 0.001).

### Correlation and classification efficacy

Within the overall study population (*N* = 111), the AI drawing score correlated significantly with both the 6-point (*ρ* = 0.638) and 10-point (*ρ* = 0.612) manual scales (all *P* < 0.001).

When stratified by cohort, the AI drawing score in the HC group maintained significant correlations with both manual scales (all *P* < 0.001). In the AD group, the AI drawing score correlated with the 6-point manual CDT (*ρ* = 0.283, *P* = 0.036), but its association with the 10-point manual CDT did not reach statistical significance (*ρ* = 0.232, *P* = 0.089).

Individual test outcomes were cross-referenced using the established cut-off threshold. The AI-based drawing platform classified 14 participants within the HC cohort (25.0%) as ‘at-risk’ (≤27.00). However, all 14 of these individuals simultaneously obtained maximum or unimpaired cognitive scores on both the traditional 6-point Shulman and 10-point Sunderland manual CDTs.

### Multivariable predictors of AD status

A multivariable binary logistic regression model was constructed with clinical diagnosis (AD vs. HC) as the dependent variable, and age, gender, and the AI drawing score as independent predictors.

The model demonstrated an excellent overall fit (McFadden's pseudo-*R*^2^ = 0.661, Likelihood Ratio test *P* < 0.001). After full demographic adjustment, the AI drawing score remained a powerful, independent predictor of AD status (*P* = 0.003; Table 3). Specifically, each 1-point decrease in the AI drawing score was associated with a 6% increase in the likelihood of a clinical AD diagnosis (OR: 0.940, 95% CI: 0.904–0.979). Age was also confirmed as a significant independent predictor (OR: 1.325, *P* < 0.001), while gender did not reach statistical significance in the adjusted model (*P* = 0.136).

**Table 3:** Multivariable logistic regression analysis predicting Alzheimer's disease Status.

Independent variables	Coefficient (*β*)	SE	Wald *z*	*P*-value	OR	95% CI
AI drawing score	−0.061	0.020	−3.02	**0.003**	0.940	[0.904–0.979]
Age	0.282	0.061	4.60	**<0.001**	1.325	[1.176–1.495]
Gender (male)	−1.139	0.764	−1.49	0.136	0.320	[0.072–1.430]
Intercept	−19.718	4.589	−4.30	**<0.001**	—	—

McFadden's pseudo-*R*^2^ = 0.661, Likelihood Ratio test *P* < 0.001. Dependent variable encoding: 0 = Healthy Control, 1 = Alzheimer's Disease. Statistically significant *P*-values are highlighted in bold.

AI, artificial intelligence, CI, confidence interval, OR, odds ratio.

## Discussion

### Principal findings

This study demonstrated that an AI-based drawing platform can effectively differentiate patients with AD from HCs with high diagnostic accuracy. The AI drawing score showed clear discriminative power, matching the overall classification performance of the traditional 10-point manual CDT scale. Furthermore, multivariable logistic regression confirmed that the AI drawing score is a significant, independent predictor of AD status, even after adjusting for substantial baseline demographic differences such as age and gender. Most notably, the AI-based drawing platform identified a significant subset of clinically asymptomatic HCs, who scored perfectly on traditional manual scales, as being ‘at-risk’, while demonstrating a divergent correlation pattern within the cognitively impaired cohort.

### Interpretation and comparison with literature

The most compelling finding of this study is that the AI-based drawing platform classified a substantial proportion (25.0%) of the HC cohort as ‘at-risk’ based on the optimal cut-off threshold (≤27.00) identified in this study, despite these individuals obtaining unimpaired or maximum scores on both the 6-point Shulman and 10-point Sunderland manual scales [17, 18]. This high rate of preclinical detection in clinically asymptomatic individuals aligns with recent digital screening literature, where advanced digital tools consistently uncover subtle cognitive deficits in populations misclassified as unimpaired by conventional instruments [20]. Traditional manual CDTs are inherently product-based and categorical; they evaluate the final, static drawing and are often insensitive to early, subclinical cognitive or motor changes, frequently limiting their utility in detecting early-stage neurodegenerative shifts [6, 7]. This diagnostic blind spot is supported by a study (>13 000 older adults), confirming traditional paper CDTs poorly identify MCI [21].

In contrast, digital drawing platforms capture fine-grained spatial and geometric features that are completely invisible to the human eye [16]. By registering these minute variations, digital drawing platforms effectively quantify intricate graphomotor patterns and micro-behavioural indicators that remain entirely undetected during conventional pen-and-paper clinical assessments [22]. These digital biomarkers provide a dynamic evaluation of executive functions regulated by subcortical and hippocampal networks. Consequently, they offer a sensitive early warning framework for preclinical cognitive decline long before overt symptoms appear [23, 24]. Advanced AI models can reveal specific structural and morphological anomalies in early drawing profiles. Conventional scoring criteria often fail to capture these nonlinear changes linearly [25]. Our results support the view that digital biomarkers can detect preclinical cognitive decline or subtle neurodegenerative signs years before standard screening rubrics register a failed score.

In terms of overall diagnostic accuracy, the AI drawing score and the 10-point scale demonstrated identical performance (AUC = 0.868), closely following the manual 6-point scale (AUC = 0.910) [17, 18]. This classification capability underscores the historical validity of the manual CDT as an excellent instrument for screening cognitive decline [1, 2], while demonstrating that AI-driven metrics can successfully match this established diagnostic precision [15]. Our diagnostic precision matches Tafiadis *et al*. [26], confirming modern CDTs yield high diagnostic power (AUC ≤ 0.899) by leveraging visuospatial construction abilities. While global evidence from recent meta-analyses underscores the general diagnostic accuracy of digital CDTs in AD screening, this AI-based drawing platform offers an alternative, pattern-driven approach that operates independently of traditional clock-face structural and semantic constraints [27]. Similarly, machine learning models combining dynamic processes and static drawing results on electronic tablets have proven highly accurate in screening for cognitive decline, demonstrating that process-related features are highly predictive of impairment across specific cognitive and motor domains [28].

The application of this AI-based drawing platform provides a practical advantage by replacing subjective scoring variability with a continuous, objective metric ranging from 0 to 100. Such automated digital approaches have been shown to optimize screening workflows and enhance objectivity in clinical environments [16]. Furthermore, implementing such AI-driven screening platforms in community-wide projects or primary healthcare settings has proven valid, feasible, and pragmatic, offering rapid administration times that substantially lower clinical burden compared to traditional paper scales [2, 14, 29].

Our correlation analysis revealed another nuanced insight into how the AI-based drawing platform evaluates advanced cognitive decline. While the AI drawing score correlated strongly with both manual scales across the entire population and within the HC cohort, its association with the 10-point Sunderland scale did not reach statistical significance within the AD group (*P* = 0.089) [18]. This divergence suggests a potential saturation or a limited sensitivity of the traditional 10-point manual scale in manifest dementia cases. In advanced stages, widespread cognitive decline leads to global drawing errors that conventional product-based scoring cannot differentiate linearly. Conversely, by utilizing continuous scoring rather than static categorical tallies, the AI-based drawing platform can capture subtle variations in cognitive-motor impairment that escape traditional point-deduction rules.

Final multivariable regression analysis underscored the independent predictive validity of the AI-based drawing platform. Although the AD cohort was significantly older than the HC cohort, reflecting the real-world epidemiology of the disease, the AI drawing score remained a significant predictor (*P* = 0.003) after demographic adjustment. Each 1-point decrease in the AI score was independently associated with a 6% increase in the odds of an AD diagnosis, confirming that the digital features extracted by the platform reflect true pathological cognitive impairment rather than merely capturing age-related motor slowing.

### Strengths and limitations

A major strength of this study is the direct, head-to-head comparison of an advanced AI-based drawing platform against two distinct, globally validated manual CDT scoring systems within a well-characterized clinical sample. By extracting objective, culture-independent kinematic and behavioural parameters, focusing purely on the drawing process rather than learned schematic representations, the AI-based drawing platform operates as an equitable screening tool that mitigates demographic, linguistic, and educational biases [16, 20]. Furthermore, by utilizing a continuous scoring system, this approach completely eliminates observer bias and inter-rater variability, making it highly scalable for digital health, remote monitoring, and primary care screening.

Leaning on the STROBE framework, several limitations must be acknowledged. First, due to the cross-sectional design of the study, we cannot longitudinally track the 14 HC participants flagged as ‘at-risk’ by the AI-based drawing platform to confirm whether they eventually progress to MCI or AD. Whether these 14 cases reflect true subclinical cognitive shifts or automated false-positives remains uncertain. Consequently, rather than receiving immediate clinical diagnoses, these individuals would benefit from closer follow-up or further assessments.

Second, there was a significant age imbalance between the two cohorts; although this was strictly controlled for in our multivariable regression model, future validation studies should ideally utilize age-matched cohorts. To address demographics, our tool uses a ‘free-drawn’ format. Caffarra *et al*. [30] showed free-drawn tasks resist normal ageing effects versus pre-drawn templates. Additionally, domain-isolated visual testing avoids mixed attention or language confounders [31–33]. Unmatched baseline age remains a limitation.

Additionally, while digital cognitive screening tools are highly feasible for primary care, literature notes that technological adaptability and user preference can vary among older populations, particularly those with lower education levels, which represents an implementation challenge to be addressed in future designs [29]. Importantly, our study lacked an independent external validation cohort, and our exploratory cut-off (<27.00) derived via Youden's Index requires prospective validation in broader independent cohorts. Finally, our platform analyses only the final static image; future versions should track dynamic line kinetics like speed or pressure. Lastly, our study lacked advanced biomarkers (such as cerebrospinal fluid amyloid/tau profiles or amyloid PET imaging) to definitively confirm the underlying biological pathology of the participants, particularly those in the preclinical ‘at-risk’ window. Additionally, our study evaluated only AD; however, Heinik *et al*. [34] showed drawing tasks can differentiate AD from vascular dementia. Testing other dementia phenotypes represents a key future step.

## Conclusion

This study demonstrates that an AI-based drawing platform provides an objective and accessible tool for screening AD. The platform matches the classification performance of traditional manual scales. Crucially, it also identifies a subset of clinically asymptomatic individuals as ‘at-risk’. These findings highlight the clinical value of continuous, kinematic features over static, product-based scores.

The platform offers rapid administration and eliminates inter-rater variability. It is also resistant to demographic, linguistic, and educational factors. These characteristics necessitate further multi-centre longitudinal validation rather than immediate implementation in clinical workflows.

Future longitudinal studies should incorporate gold-standard fluid or neuroimaging biomarkers. This research is needed to track the clinical progression of individuals identified in the preclinical window. Ultimately, it will help define the platform's role in early diagnostic and therapeutic pathways.

## Supplementary Material

cmag066_Supplementary_Data

## Data Availability

The data underlying this article will be shared at reasonable request to the corresponding author.
