# Supplementary material for: Validation of an artificial intelligence-based drawing platform against the Clock Drawing Test for cognitive screening: a comparative study between Alzheimer's disease and healthy controls
Source: Fam Pract. 2026 Aug 25;43(5):cmag066. doi: 10.1093/fampra/cmag066 (PMC13504279; doi:10.1093/fampra/cmag066)
Supplement: Supplementary file 1 [file cmag066_supplementary_data.pdf]

The STARD reporting checklist

For checking that diagnostic accuracy study articles can be understood and used by everyone

How to use this reporting checklist

This reporting checklist allows authors to demonstrate that their manuscripts adhere to the STARD reporting guideline.

If you have not used a reporting guideline before, read about how and why to use them and check whether STARD is the most applicable reporting guideline for your work.

Reporting guidelines are most useful when used early in research. When writing a manuscript or application, consider using the full guidance where you'll find explanations and examples for each item.

After writing, demonstrate adherence by completing this checklist:

1. Specify where each item is described (see Note 1).
2. Cite this checklist (See Note 2).
3. Include your completed checklist as a supplement when submitting to a journal so that future readers can use it to find information.

	Item Description	Location (or reason for not reporting)
**Title or abstract**		
1. Identification as a study of diagnostic accuracy	Identification as a study of diagnostic accuracy using at least one measure of accuracy (such as sensitivity, specificity, predictive values, or AUC).	Title (page 1) and Abstract (page 3), Methods (page 6) and Results (page 10).
**Abstract**		
2. Abstract	Structured summary of study design, methods, results and conclusions (for specific guidance, see STARD for Abstracts).	Abstract (page 3).
**Introduction**		
3. Background	Scientific and clinical background, including the intended use and clinical role of the index test.	Introduction, paragraphs 1, 2, and 3 (page 4).
4. Objectives	Study objectives and hypotheses.	Introduction, paragraph 4 and 5 (page 4, 5).
**Methods**		
5. Study design	Whether data collection was planned before the index test and reference standard were performed (prospective study) or after (retrospective study).	Methods, "Study Design and Setting" section, paragraph 1 (page 6); explicitly stated as a cross-sectional,

		methodological validation study.
Participants		
6. Eligibility criteria	Eligibility criteria.	Methods, "Participants and Selection Criteria" section, paragraphs 1 and 2 (pages 6 and 7).
7. Identifying eligible participants	On what basis potentially eligible participants were identified (such as symptoms, results from previous tests, inclusion in registry).	Methods, "Sampling Frame and Target Population" section, paragraph 1 (page 6); based on ICD-10 codes in the primary care database and nursing home residence.
8. Setting, location, and dates	Where and when potentially eligible participants were identified (setting, location and dates).	Methods, "Study Design and Setting" (page 6) and "Sampling Frame and Target Population" (page 6). Tuzla and Maltepe, Nov 1, 2023 - Feb 1, 2024.
9. Consecutive, random or convenience series	Whether participants formed a consecutive, random or convenience series.	Methods, "Group Allocation" section, paragraph 1 (page 6); total population sampling for AD and convenience sampling for HCs.
Test Methods		
10. Index test & Reference standard	10a. Index test<br>10b. Reference standard	10a. Methods, "Data Collection and Assessment Tools" section (page 7); AI-Based Drawing Assessment on a tablet using a digital pen.<br><br>10b. Methods, "Data Collection and Assessment Tools" section (page 7); Traditional CDT scored via Shulman (6-point) and Sunderland (10-point) systems, linked with clinical diagnosis.
11. Reference standard rationale	Rationale for choosing the reference standard (if alternatives exist).	Introduction, paragraph 1 (page 4).
12. Index test and reference standard cut-offs or categories	12a. Definition of and rationale for test positivity cut-offs or result categories of the index test, distinguishing prespecified from exploratory.	12a. Methods, "Statistical Analysis" section, paragraph 1 (page 8) and Results, "Diagnostic Accuracy and ROC Curve Analysis" section,

	12b. Definition of and rationale for test positivity cut-offs or result categories of the reference standard, distinguishing prespecified from exploratory.	paragraph 2 (page 10); cut-off was exploratory, determined using Youden's Index.<br><br>12b. Methods, "Data Collection and Assessment Tools" section (page 7); prespecified global standard scoring scales (Shulman 6-point and Sunderland 10-point).
13. Information available to performers or readers of the index test and reference standard assessors	13a. Whether clinical information and reference standard results were available to the performers or readers of the index test.<br><br>13b. Whether clinical information and index test results were available to the assessors of the reference standard.	13a. Not applicable; the index test score was entirely automated and calculated by an offline pre-trained Convolutional Neural Network (CNN) architecture, preventing human observer bias.<br><br>13b. Methods, "Data Collection and Assessment Tools" section (page 7); drawings were independently evaluated using manual scales to minimize scoring bias.
Analysis		
14. Analysis methods	Methods for estimating or comparing measures of diagnostic accuracy.	Methods, "Statistical Analysis" section, paragraph 1 and 2 (page 8); ROC curve analysis, AUC with bootstrapped 95% CIs, Youden's Index, and Cohen's Kappa.
15. Indeterminate results	How indeterminate index test or reference standard results were handled.	No indeterminate results occurred; all included participants successfully completed both the manual and digital protocols as per the total population/convenience framework.
16. Missing data	How missing data on the index test and reference standard were handled.	No missing data; completion of both evaluation protocols was required for final sample inclusion (N = 111).
17. Variability	Any analyses of variability in diagnostic accuracy, distinguishing prespecified from exploratory.	Methods, "Statistical Analysis" section (page 8) and Results

		section (page 10, 11); exploratory stratification by cohort (AD vs. HC) was performed during correlation analysis.
18. Intended sample size	Intended sample size and how it was determined.	Methods, "Sample Size and Power Justification" section, paragraph 1 (page 7); sample size was defined by maximum feasible enrolment within clinical settings, backed by post-hoc power analysis.
**Results**		
Participants		
19. Participant flow diagram	Flow of participants, using a diagram.	Described textually in Methods, "Group Allocation" section (page 6) and Results, paragraph 1 (page 10). No formal flow diagram was included as eligibility directly matched final sample analysis.
20. Baseline characteristics	Baseline demographic and clinical characteristics of participants.	Results, "Baseline Demographics and Clinical Characteristics" section, paragraphs 1 and 2 (page 10), and cross-referenced to Table 1.
21a. Participants with and without the target condition	21a. Distribution of severity of disease in those with the target condition.<br>21b. Distribution of alternative diagnoses in those without the target condition	21a. Results, "Baseline Demographics and Clinical Characteristics" section, paragraph 2 (page 10), and Table 1 (showing median manual CDT scoring profiles for the clinical AD cohort).<br><br>21b. Not applicable; the non-AD cohort comprised strictly cognitively healthy older adults (HCs) screened and selected from the primary care database.
22. Time interval	Time interval and any clinical interventions between index test and reference standard.	Methods, "Data Collection and Assessment Tools" section (page 7); both tests were administered in parallel during a single-session assessment.

Test Results		
23. Index test and reference standard results	Cross tabulation of the index test results (or their distribution) by the results of the reference standard.	Results, "Correlation and Classification Efficacy" section, paragraphs 1, 2, and 3 (page 11); textual cross-referencing and continuous distribution comparisons are detailed.
24. Estimates of accuracy	Estimates of diagnostic accuracy and their precision (such as 95% CIs).	Abstract (page 3) and Results, "Diagnostic Accuracy and ROC Curve Analysis" section, paragraphs 1 and 2 (page 10), and cross-referenced to Table 2.
25. Adverse events	Any adverse events from performing the index test or the reference standard.	No adverse events were reported or anticipated, as both index and reference tests are non-invasive, digital/paper-based cognitive screening tasks.
**Discussion**		
26. Limitations	Study limitations, including sources of potential bias, statistical uncertainty and generalisability.	Discussion, "Strengths and Limitations" section, paragraph 2, 3 and 4 (page 15 and 16).
27. Implications for Practice	Implications for practice, including the intended use and clinical role of the index test.	Abstract (page 3), Discussion ("Principal Findings" and "Interpretation" sections, page 12-15), and Conclusion (page 16 and 17).
**Other information**		
28. Registration	Registration number and name of registry.	None; this was an observational, non-interventional methodological validation study and did not undergo clinical trial registration.
29. Protocol	Where the full study protocol can be accessed.	The study protocol is not publicly registered; however, detailed procedural frameworks are fully disclosed within the Methods section.
30. Funding	Sources of funding and other support; role of funders.	Declarations, "Funding" section (page 17); authors declare no funding support.

1 How to specify where content is

Tell the reader where they can find information. E.g.,

- Results; paragraph 2
- Methods, Participants; paragraphs 1 & 2.
- Table 3
- Supplement B, para. 4

If you have chosen not to describe an item, explain why. You can do this in the checklist, or as a note below it.

You can describe items in the article body, or in tables, figures, or supplementary materials, and should prioritize items you feel are most important to your intended audience. The order of items in your manuscript does not need to match the order of items in this checklist. You can decide how best to structure your work.

2 How to cite

Describe how you used STARD at the end of your Methods section, referencing the resources you used e.g.,

> 'We used the STARD reporting guideline(1) to draft this manuscript, and the STARD reporting checklist(2) when editing, included in supplement A'

If you use a reporting checklist, remember to include it as a supplement when publishing so that readers can easily find information and see how you have interpreted the guidance.

1. Bossuyt PM, Reitsma JB, Bruns DE, Gatsonis CA, Glasziou PP, Irwig L, et al. STARD 2015: An updated list of essential items for reporting diagnostic accuracy studies. BMJ [Internet]. 2015 Oct;351:h5527. Available from: https://www.bmj.com/content/351/bmj.h5527

2. Bossuyt PM, Reitsma JB, Bruns DE, Gatsonis CA, Glasziou PP, Irwig L, et al. The STARD reporting checklist. In: Harwood J, Albury C, Beyer J de, Schlüssel M, Collins G, editors. The EQUATOR network reporting guideline platform [Internet]. The UK EQUATOR Centre; 2025. Available from: https:/resources.equator-network.org/reporting-guidelines/stard/stard-checklist.docx
